# Estimating the Replicability of Sports and Exercise Science Research

**DOI:** 10.1007/s40279-025-02201-w

**Published:** 2025-06-16

**Authors:** Jennifer Murphy, Aaron R. Caldwell, Cristian Mesquida, Aera J. M. Ladell, Alberto Encarnación-Martínez, Alexandre Tual, Andrew Denys, Bailey Cameron, Bas Van Hooren, Ben Parr, Bianca DeLucia, Billy R. J. Mason, Brad Clark, Brendan Egan, Calum Brown, Carl Ade, Chiarella Sforza, Christopher B. Taber, Christopher Kirk, Christopher McCrum, Cian OKeeffe Tighe, Ciara Byrne, Claudia Brunetti, Cyril Forestier, Dan Martin, Danny Taylor, David Diggin, Dearbhla Gallagher, Deborah L. King, Elizabeth Rogers, Eric C. Bennett, Eric T. Lopatofsky, Gemma Dunn, Gérome C. Gauchard, Guillaume Mornieux, Ignacio Catalá-Vilaplana, Ines Caetan, Inmaculada Aparicio-Aparicio, Jack Barnes, Jake Blaisdell, James Steele, Jared R. Fletcher, Jasmin Hutchinson, Jason Au, Jason P. Oliemans, Javad Bakhshinejad, Joaquin Barrios, Jose Ignacio Priego Quesada, Joseph Rager, Julia B. Capone, Julie S. J. Walton, Kailey Stevens, Katie Heinrich, Kelly Wu, Kenneth Meijer, Laura Richards, Lauren Jutlah, Le Tong, Lee Bridgeman, Leo Banet, Leonard Mbiyu, Lucy Sefton, Margaux de Chanaleilles, Maria Charisi, Matthew Beerse, Matthew J. Major, Maya Caon, Mel Bargh, Michael Rowley, Miguel Vaca Moran, Nicholas Croker, Nicolas C. Hanen, Nicole Montague, Noel E. Brick, Oliver R. Runswick, Paul Willems, Pedro Pérez-Soriano, Rebecca Blake, Rebecca Jones, Rebecca Louise Quinn, Roberto Sanchis-Sanchis, Rodrigo Rabello, Roisin Bolger, Roy Shohat, Sadie Cotton, Samantha Chua, Samuel Norwood, Samuel Vimeau, Sandro Dias, Sissel Pedersen, Spencer S. Skaper, Taylor Coyle, Terun Desai, Thomas I. Gee, Tobias Edwards, Torsten Pohl, Vanessa Yingling, Vinicius Ribeiro, Youri Duchene, Zacharias Papadakis, Joe P. Warne

**Affiliations:** 1 School of Biological, Health and Sport Sciences, Technological University Dublin, Tallaght Campus, Blessington Rd, Dublin 9, Ireland; 2 Institute for Community Health Innovation, University of Arkansas for Medical Sciences Northwest, Springdale, AR 72762 USA; 3 Human-Technology Interaction Group, Eindhoven University of Technology, Eindhoven, The Netherlands; 4 Department of Health and Physical Education, Mount Royal University, Calgary, AB T3E 6K6 Canada; 5 Department of Physical Education and Sports, Universitat de València, València, Spain; 6 Laboratoire Motricité, Interactions, Performance, Le Mans Université, Le Mans, France; 7 Department of Kinesiology, California State University East Bay, Hayward, CA 94542 USA; 8 Department of Sport and Health, Solent University, Southampton, SO14 0YN UK; 9 Department of Nutrition and Movement Sciences, NUTRIM School for Nutrition and Translational Research in Metabolism, Maastricht University, 6211 LK Maastricht, The Netherlands; 10 School of Psychology, Ulster University, Coleraine, Co., Londonderry, BT52 1SA UK; 11 Department of Exercise Science and Athletic Training, Springfield College, Springfield, MA USA; 12 University of Canberra Research Institute for Sport and Exercise, University of Canberra, Bruce, ACT 2617 Australia; 13 School of Health and Human Performance, Dublin City University, Glasnevin, Dublin 9, Ireland; 14 School of Sport and Physical Activity, Sheffield Hallam University, Sheffield, UK; 15 Department of Kinesiology, Kansas State University, Manhattan, KS 66506 USA; 16 Department of Biomedical Sciences for Health, Università Degli Studi Di Milano, Via Mangiagalli, 31, 20133 Milan, Italy; 17 Department of Physical Therapy and Human Movement Science, Sacred Heart University, Fairfield, CT USA; 18 School of Sport and Exercise Science, University of Lincoln, Lincoln, LN6 7TS UK; 19 School of Health Sciences and Human Performance, Ithaca College, Ithaca, NY 14850 USA; 20 School of Human and Social Sciences, Buckinghamshire New University, High Wycombe, UK; 21 Development, Adaptation, Handicap-CARE Grand Est, Université de Lorraine, Nancy, France; 22 Department of Physical Therapy, The University of British Columbia, Vancouver, Canada; 23 Department of Kinesiology and Health Sciences, University of Waterloo, Waterloo, ON Canada; 24 Department of Physical Therapy, University of Dayton, Dayton, OH USA; 25 Institute of Psychiatry, Psychology and Neuroscience, Kings College London, London, UK; 26 Faculty of Kinesiology, University of Calgary, Calgary, AB T2N 1N4 Canada; 27 Department of Physical Medicine & Rehabilitation, Northwestern University, Chicago, IL USA; 28 Human Performance Laboratory and Motion Analysis Center, Department of Health Promotion and Clinical Practice, Barry University, Miami Shores, FL 33161 USA; 29 Specialist Facilities, Solent University, Southampton, SO14 0YN UK; 30 Institute of Sport, Exercise and Health, Division of Surgery and Interventional Science, University College London, London, UK; 31 Conservative and Rehabilitative Orthopaedics, Technical University of Munich, G80992 Munich, Germany

## Abstract

**Background:**

The replicability of sports and exercise research has not been assessed previously despite concerns about scientific practices within the field.

**Aim:**

This study aims to provide an initial estimate of the replicability of applied sports and exercise science research published in quartile 1 journals (SCImago journal ranking for 2019 in the Sports Science subject category; www.scimagojr.com) between 2016 and 2021.

**Methods:**

A formalised selection protocol for this replication project was previously published. Voluntary collaborators were recruited, and studies were allocated in a stratified and randomised manner on the basis of equipment and expertise. Original authors were contacted to provide deidentified raw data, to review preregistrations and to provide methodological clarifications. A multiple inferential strategy was employed to analyse the replication data. The same analysis (i.e. *F* test or *t* test) was used to determine whether the replication effect size was statistically significant and in the same direction as the original effect size. *Z*-tests were used to determine whether the original and replication effect size estimates were compatible or significantly different in magnitude.

**Results:**

In total, 25 replication studies were included for analysis. Of the 25, 10 replications used paired *t* tests, 1 used an independent *t* test and 14 used an analysis of variance (ANOVA) for the statistical analyses. In all, 7 (28%) studies demonstrated robust replicability, meeting all three validation criteria: achieving statistical significance (*p* < 0.05) in the same direction as the original study and showing compatible effect size magnitudes as per the *Z* test (*p* > 0.05).

**Conclusion:**

There was a substantial decrease in the published effect size estimate magnitudes when replicated; therefore, sports and exercise science researchers should consider effect size uncertainty when conducting subsequent power analyses. Additionally, there were many barriers to conducting the replication studies, e.g., original author communication and poor data and reporting transparency.

## Key Points

**Table:** 

This is the first large replication project in sports and exercise science due to concerns about replication in the field.
Findings showed that 28% of studies were replicated successfully.
The results raise concerns about research findings in this discipline that should be explored further.
Researchers should consider the accumulation of evidence rather than relying on standalone findings in sports and exercise science.

## Introduction

Science is a dynamic process of discovering new effects and testing theories and their application. Yet, an “effect” that has been found once but cannot be replicated arguably does not qualify as a scientific discovery [1]. Replication studies are at the heart of the scientific process, as they advance knowledge by confirming or refuting previous findings or explore boundary conditions and the underlying variation in the true effect [2]. Although it is generally accepted that replications play an important role in science, the inability to reproduce research findings is still a long-standing problem [3]. The current research culture is largely driven by career incentives and novel research, which has given rise to poor scientific behaviours that prioritise individual researcher success (e.g. career advancement) rather than what is beneficial for science as a whole [4–6]. This motivates researchers to place emphasis on novel or “flashy” findings and hunt for statistical significance within their datasets to garner a “high impact” publication. Consequently, replication has come to the forefront of discussions, particularly in psychology, due to the observed failures to replicate well-known psychological results [7]. This exacerbated the “crisis of confidence” in scientific findings [8, 9], and there is also evidence of scientific misconduct, including fraud and questionable research practices e.g., *p*-hacking, multiple analyses and selective reporting of a desirable result [10–12]. The proclamation that most research findings are false has fuelled scepticism within the scientific community, prompting the adoption of open science practices to enhance transparency and rigour [13].

Sports and exercise science, similar to other fields, has grappled with criticisms surrounding questionable research practices, including overly optimistic statistical conclusions and a scarcity of replication studies [14]. Overall, there are concerns about study design, and statistical and reporting practices within the field, resulting in calls for more replication studies [15, 16]. A history of low-powered studies has contributed to these concerns [17], as well as the rampant misuse of null hypothesis significance testing (NHST) [18–20]. In particular, stating the null and alternative hypotheses and setting both the alpha and beta levels are prerequisites for NHST. Still, investigations show less than a quarter of studies report an a priori power analysis and 82% of studies that do not state a hypothesis use NHST anyway [20–22]. Reporting transparency is also an obvious problem in sports and exercise science, with a data sharing rate of less than 5% and almost zero studies making computer code available [23]. Furthermore, implausibly high positive result rates of 81% across sports and exercise science journals [20], and 82% across sports medicine and physiotherapy journals [24], indicate the presence of publication bias given the average observed power of studies [22, 25]. Sports and exercise scientists also rarely collaborate with statisticians [26], despite the regularity of statistical errors [27] and our awareness of our overall sub-standard statistical competency [28]. These errors are then compounded by further errors in meta-analyses [29]. Finally, the reporting of basic statistical information such as test statistics, degrees of freedom and confidence intervals is sporadic at best [22]. All of these issues raise concerns about replicability within our field.

The aforementioned concerns, coupled with limited reporting of null or trivial results and a lack of transparency, underscore the need for a reformation of research practices within sports and exercise science [15]. A subjective survey of over 500 sports and exercise scientists revealed a widespread belief in a reproducibility (using the same data to obtain the same results) and replicability (using new data to obtain a similar result) crisis within the field, and despite ongoing discussions about these issues, substantial barriers to both reproducibility and replicability persist [28]. While isolated replication studies exist [30–33], a comprehensive quantitative assessment of replicability in our field remains elusive. Most discussions on the topic rely on indirect inferences or anecdotes rather than empirical data. Thus, this study aims to provide an initial estimate of the replicability of sports and exercise science research published in quartile 1 journals between 2016 and 2021. Previous research reports that the expected replication rate was 0.61, suggesting that, if we replicate significant findings (with the same statistical power and sample size), 61% would be expected to yield another significant effect [22]. This was a pilot study of 89 studies published in the *Journal of Sport Science* and requires further research; however, in the absence of any other information, we expect a similar replication rate here.

## Methods

### Replication Study Selection

This project began in September 2020 with the completion of data collection in 2024. A formalised selection protocol for this replication project was created to minimise bias where possible and published for full transparency [34]. The key aspects of the selection protocol focused on the year of publication and citation rankings, research discipline, study type, the research question and key dependent variable, study methods and feasibility. In summary, studies were selected if they had a statistically significant main effect published between 2016 and 2021 in quartile 1 applied sports and exercise science journals (SCImago journal rank for 2019 in the Sports Science subject category; www.scimagojr.com) and they were experimental or quasi-experimental quantitative studies, whereby an independent variable was manipulated to determine the effect on the dependent variable, in pairwise, independent or crossover study designs and across two or more groups. The project leaders selected the key applied dependent variable that was first stated in the abstract, first or primary hypothesis or aim. If this dependent variable was not an applied sports and exercise science variable, or if it was not analysed using a *t*-test or analysis of variance (ANOVA) as per the selection criteria [34], this dependent variable was disregarded, and the next stated applied dependent variable was selected from the abstract, first or primary hypothesis or aim. Where this was unclear, the dependent variable was randomly selected when it met all of the other inclusion criteria. Studies to be replicated were screened by J.M., J.W. and C.M. using an online survey which was created to screen potential replication studies (https://doi.org/10.17605/OSF.IO/SFBVA). When the screening was completed, the studies were divided into research sub-disciplines (applied sports and exercise biomechanics, psychology, physiology, nutrition and injury prevention) and numbered (https://doi.org/10.17605/OSF.IO/SFBVA).

### Collaborator Recruitment and Allocation

Voluntary collaborators were recruited via social media, where researchers filled out an expression of interest form, including details of expertise, equipment, software and their versions in their laboratories. Collaborators were then matched to a replication study when the equipment was readily available (e.g., Optojump™) and the study topic was in the researcher’s area of expertise. If more than one study was matched to a collaborator, one study was allocated using a random number generator. Allocation was completed by J.M. only. Collaborators then reviewed their allocated study for feasibility and accepted or rejected the study. Feasibility checks included reviewing the type and number of participants to be recruited (i.e., could they access the population?), access to equipment and availability of laboratory hours at their university, etc. We emphasised that rejection was only possible based on feasibility and not because of personal preference for any study type or topic, but this required faith and trust in our collaborators to adhere to this. The process of study allocation was repeated if a different study was needed. Only a subset of studies from the larger study pool could be replicated in line with the number of collaborators who volunteered for this project. Ethical approval was obtained at each local university.

### Replication Study Preparation

#### Contacting of Original Author

As we aimed to conduct close replications, methods for the replication study were based closely on the original study, with any differences being those that were unavoidable e.g., a new sample or different equipment [35]. When a study was a potential match, the corresponding original author was contacted to inform them of a potential replication attempt and to maximise replication quality. At this stage, we requested the deidentified raw data for the specific dependent variable of interest and any other materials deemed important to the replication e.g., statistical code. Another author was contacted (the last author) where possible if the corresponding author did not respond. Later, when the study was accepted for replication by the collaborator, we contacted the original authors again if raw data had not been provided to ask for full details of test statistics where they were not reported (e.g., *t*-values or *F*-values, degrees of freedom and exact *p*-values) and further methodological details where necessary. Each replication study was also individually preregistered on the *Open Science Framework* (overall project page: https://doi.org/10.17605/OSF.IO/3VUFG). We contacted the original authors again to provide them with an opportunity to review or express concerns about the preregistration. Any concerns from the original authors were discussed amongst the replication team and applied to the replication study where possible while trying to maximise replication quality and minimise deviation from the original published protocols. Our collaborating researchers were entitled to publish the individual replication study as an independent study and were required to contact the original authors to provide them with an opportunity to review the manuscript before submission.

#### Statistical Power Calculations

Multiple methods for sample size calculation were used and are fully detailed in the formalised selection protocol [34]. Briefly, we aimed to adjust for uncertainty around the original effect size point estimate and potential publication bias by using the *BUCSS R* package [36, 37]. However, this method was not always possible, as it does not give an output (infinite) if publication bias is deemed too high. As a result, we also calculated the replication sample size using the observed effect size from the original study or the lower limit of the observed effect size confidence interval at power ≥ 95%. Lastly, if the other methods could not be used, or the replication sample size was calculated as smaller than the original sample size, the original sample size was simply doubled. *R* files for all power analyses and sample size calculations (including justification of the method chosen) are available on the *Open Science Framework* (overall project page: https://doi.org/10.17605/OSF.IO/3VUFG). In addition, preregistrations for each replication study, along with supplementary materials, the study screening survey and a list of selected studies, are also available (https://doi.org/10.17605/OSF.IO/SFBVA).

### Data Management and Analysis

A multiple inferential strategy was employed to analyse the replication data. Despite the ongoing debates about the strengths and weaknesses of NHST as a statistical inference procedure [38–41], it remains the most commonly used procedure in sports and exercise science. Vote counting is one of the main methods to assess replication outcomes using NHST [42]; therefore, this method was used to determine whether the replication effect size was statistically significant, and in the same direction as the original effect size. In other words, the same statistical analysis was applied in the replication study as in the original study, when statistical assumptions were met (e.g., normality), at the original study’s alpha level. In cases where replication data were not normal, we visually inspected boxplots and computed the interquartile range. Any extreme outliers were removed from the dataset, and the final replication sample size (Tables 1, 2) reflects the sample size after the removal of outliers. The vote counting method can result in an exaggeration of replication failures when solely used to assess replication outcomes [42]; therefore, we also compared effect size estimates to assess the potential inflation of those estimates as a result of small sample size and bias [21, 43].

**Table 1:**
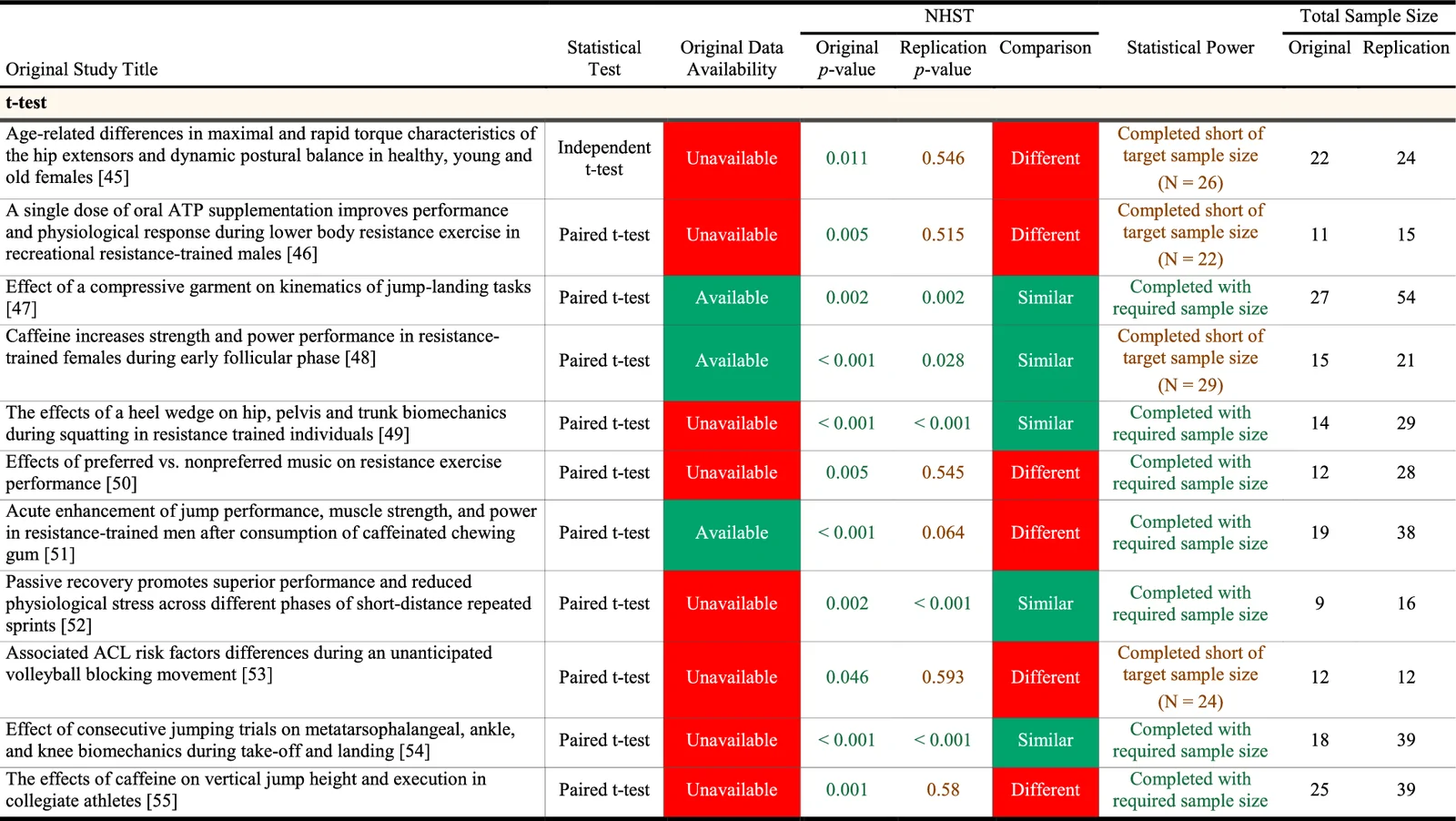
Original and replication study descriptives for the *t*-test studies

*ACL* anterior cruciate ligament, *ATP* adenosine triphosphate, *NHST* null hypothesis significant testing

**Table 2:**
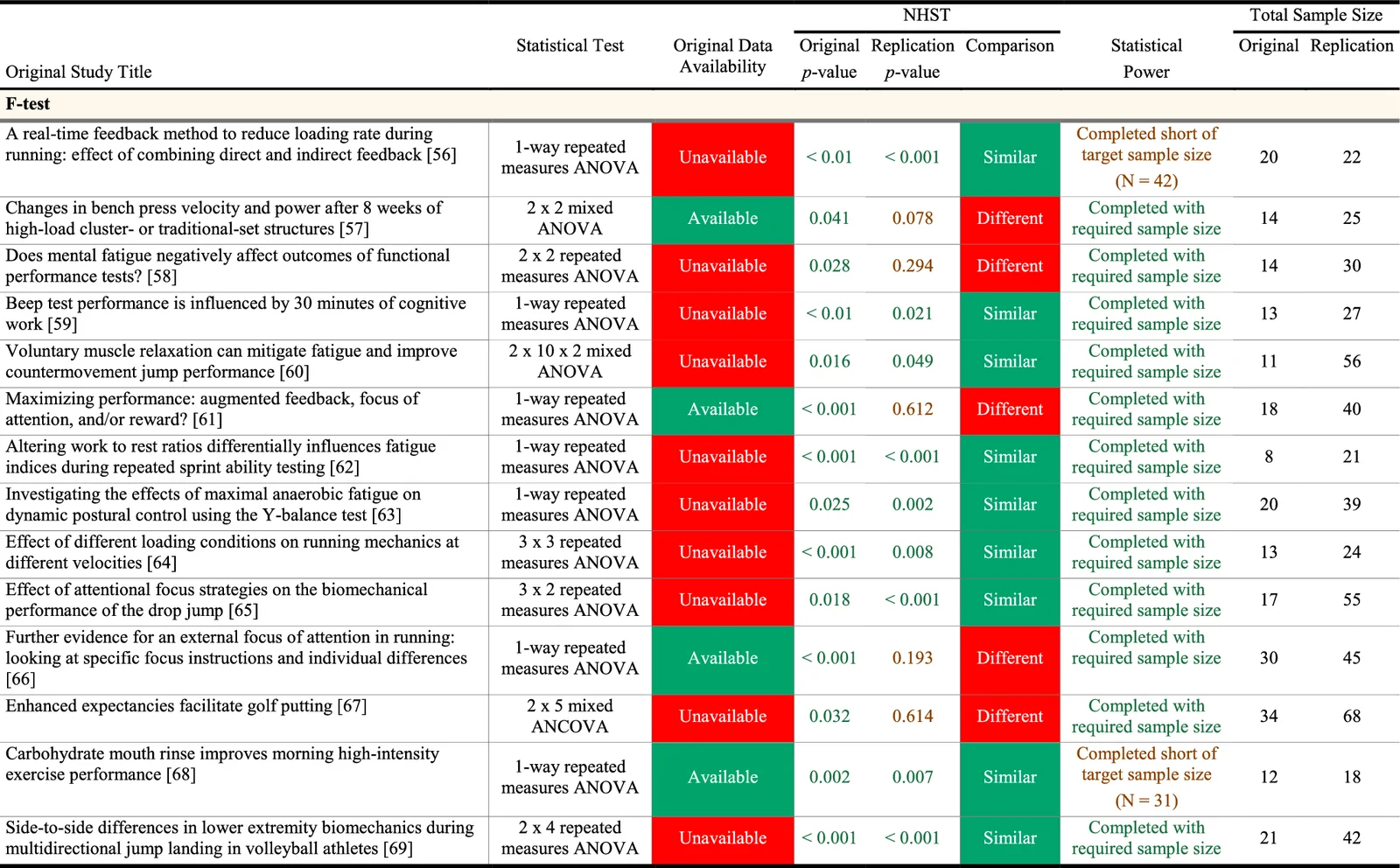
Original and replication study descriptives for the *F*-test studies

*NHST* null hypothesis significant testing

Assessing the direction of both the original and replication effect sizes is a simple technique to implement (i.e. to answer the question: are both effect size estimates in the same direction?), but quite limited for evaluating replicability. Consequently, we quantitatively compared the effect size estimates using *Z*-tests in the *TOSTER R* package (version 0.8.3) to measure compatibility between the original and replication study when the same underlying effect was being measured [44]. For this, the original and replication effect sizes were converted into a *z*-score, and a one-tailed *p*-value was computed to determine whether the original effect size was significantly larger than the replication (alpha = 0.05). In cases where the reported effect size was not appropriate for the study design (e.g., a Cohen’s *d*_*av*_ was reported instead of Cohen’s *d*_*z*_ for a paired design), or where we could not reproduce the original effect size, we calculated the effect size for the original study using the reported information (means, test statistics, sample size and degrees of freedom). We then computed the *Z*-test to compare the appropriate effect sizes (Eq. 1) e.g., the calculated original *d*_*z*_ versus the replication *d*_*z*_ but also compared the reported effect size to the appropriate replication effect size (see supplementary materials on https://doi.org/10.17605/OSF.IO/SFBVA).

1 $$z = \frac{{d_{{{\mathrm{original}}}} - d_{{{\mathrm{replication}}}} }}{{\sqrt {{\mathrm{SE}}_{{d_{{{\mathrm{original}}}} }}^{2} + {\mathrm{SE}}_{{d_{{{\mathrm{replication}}}} }}^{2} } }}.$$

In accordance with the guidelines of Brandt et al. [35], we considered a study successfully replicated when it was significantly different from the null (i.e., *p* < 0.05 for the *t*-test or *F*-test) and the effect sizes were not significantly different and were compatible (*p* > 0.05 for the *Z*-test). Otherwise, studies were classified as an informative failure to replicate (either not different from null or in the opposite direction from the original, and a replication effect size that was significantly different from the original effect size), a practical failure to replicate (both significantly different from the null and a replication effect size that was significantly different from the original effect size) or inconclusive (neither significantly different from null, and replication and original effect sizes that were not significantly different). All data, code and analyses are available online (https://doi.org/10.17605/OSF.IO/SFBVA).

Finally, we completed a post-replication recipe to report differences between the original and replication studies [35], and created tables of methodological differences between original and replication studies, all of which are available on the individual replication project pages (accessed through the overall project here: https://doi.org/10.17605/OSF.IO/3VUFG). On the basis of these, we present short notes of interest for each replication study which inform our subjective assessment of replication quality, rated as “poor”, “moderate” and “good”.

## Results

### Screening

We screened 9385 studies, and 638 abstracts met our selection criteria [34]. On further review of the full text, 587 articles were included, and 51 were excluded, as they did not meet the criteria of the selection protocol (e.g. a linear mixed model was conducted, and that was not immediately obvious from the review of the abstract). The final pool of studies was divided into sub-disciplines (applied sports and exercise biomechanics, psychology, physiology, nutrition and injury prevention), and from this stratified pool of studies, a study was randomly selected for allocation to the collaborator for replication when the equipment was readily available to them, and the discipline was matched to their expertise. Our specific methods for this selection protocol have been described previously [34].

### Completed Replications

Of 189 collaborators who expressed interest, 33 collaborators began the process of conducting replication studies. One dropped out due to equipment malfunction, one could not make the data collection deadline and two did not follow the original protocols exactly. Therefore, 29 finished data collection. Of these completed replications, 19 reached the requested sample size, and 6 were short of this sample size but greater than the original sample size. However, 4 replication sample sizes were smaller than the original sample size and were removed from the analysis. In total, 25 replications were analysed. The mean replication sample size was *n* = 33, while the mean original sample size was *n* = 17.

### Original Author Contact, Data Sharing and Reporting

We contacted a total of 156 original authors throughout the selection process for deidentified raw data when they were a potential match to collaborators. Of those, 14% (*n* = 21) shared data. Of the 29 completed replication studies, 24% (*n* = 7) of the original study authors shared data. If the original authors did not provide the raw data, we asked for test statistics; however, no response was received. We additionally contacted the original authors to provide them with an opportunity to review or express concerns about the preregistration; 31% (*n* = 9) reviewed the preregistrations, and of these, 56% (*n* = 5) approved the preregistration, and 44% (*n* = 4) expressed concerns about the replication study.

Of the 25 analysed replications, 10 used paired *t*-tests, 1 used an independent *t*-test and 14 used an ANOVA for the statistical analyses (we focused on the main effects of the factorial designs). In the original studies (*N* = 25), 48% reported the test statistic; otherwise, 16% were calculated using the data provided by the original authors, and 36% were estimated on the basis of reporting information in the original study. For the degrees of freedom, 36% were reported; otherwise, 16% were calculated using the provided data, and 48% were estimated. Lastly, 68% reported effect size point estimates, 12% were calculated using original data, and 20% were estimated.

### Replication Outcomes

As stated in our selection protocol [34], we selected original studies with statistically significant findings. For the replication NHST outcomes, 56% (*n* = 14) were significant, similar to the original studies, and 44% (*n* = 11) were not significant (Tables 1, 2).

In the 10 original studies that conducted paired *t*-tests, we calculated all of the Cohen’s *d*_*z*_, as 5 original studies reported a Cohen’s *d*_*av*_, 2 studies did not report an effect size estimate, 1 study reported a Cohen’s *d*_*s*_ and 1 could not be reproduced. One study reported a Hedges *g*, a bias-corrected effect size, but we calculated Cohen’s *d*_*z*_ for the *Z*-test comparison. We estimated the Cohen’s *d*_*s*_ for the independent *t*-test, as we could not reproduce the original reported effect size. For the ANOVAs, we calculated 6 partial eta squared, as we could not reproduce 3 that were published, and 3 did not report any partial eta squared at all (Tables 3, 4).

**Table 3:**
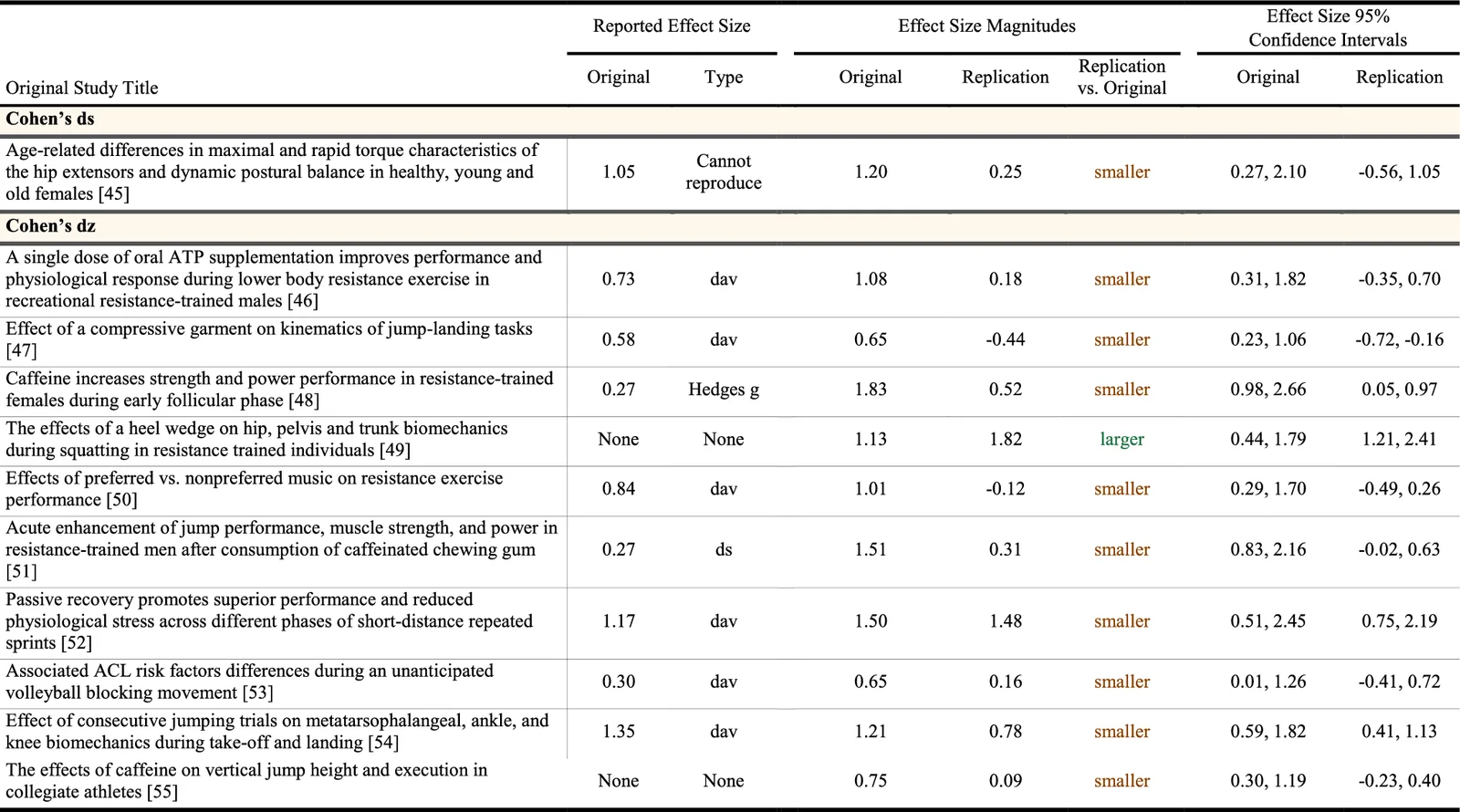
Effect size estimates (Cohen’s *d*)

Effect size magnitudes refer to the effect size estimates recalculated by the replication team. Cohen’s *d*_s_, *d*_z_ and *d*_av_ differ on the basis of the calculation of the denominator. The denominator for Cohen’s *d*_s_ is the pooled standard deviation, for Cohen’s *d*_z_ is the standard deviation of the difference scores and for Cohen’s *d*_av_ is the average standard deviation [70]

*ACL* anterior cruciate ligament, *ATP* adenosine triphosphate

**Table 4:**
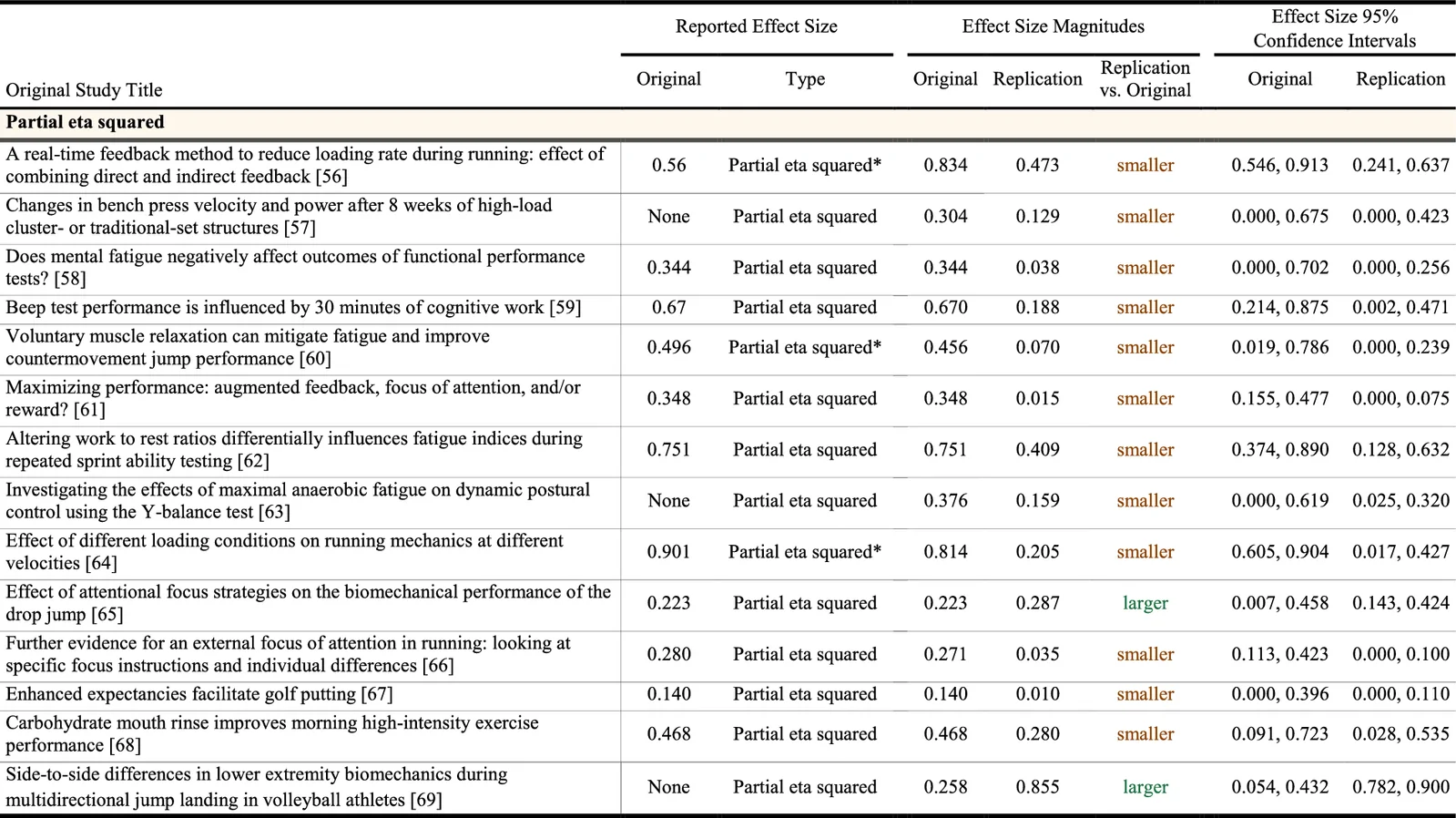
Effect size estimates (partial eta squared)

^a^Stated as partial eta squared but cannot reproduce

**Fig. 1:**
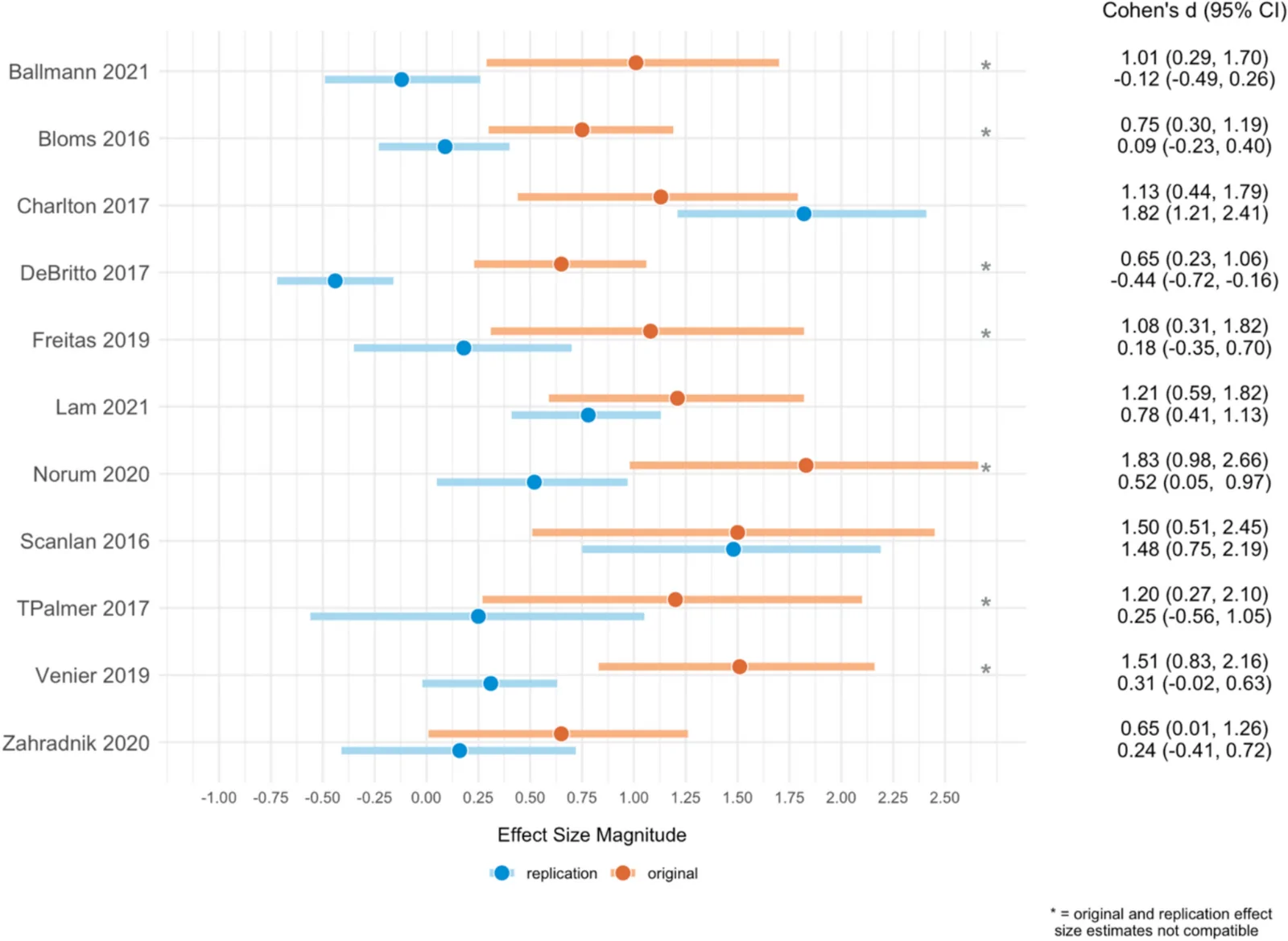
Plot showing original and replication Cohen’s *d* magnitude and confidence intervals

**Fig. 2:**
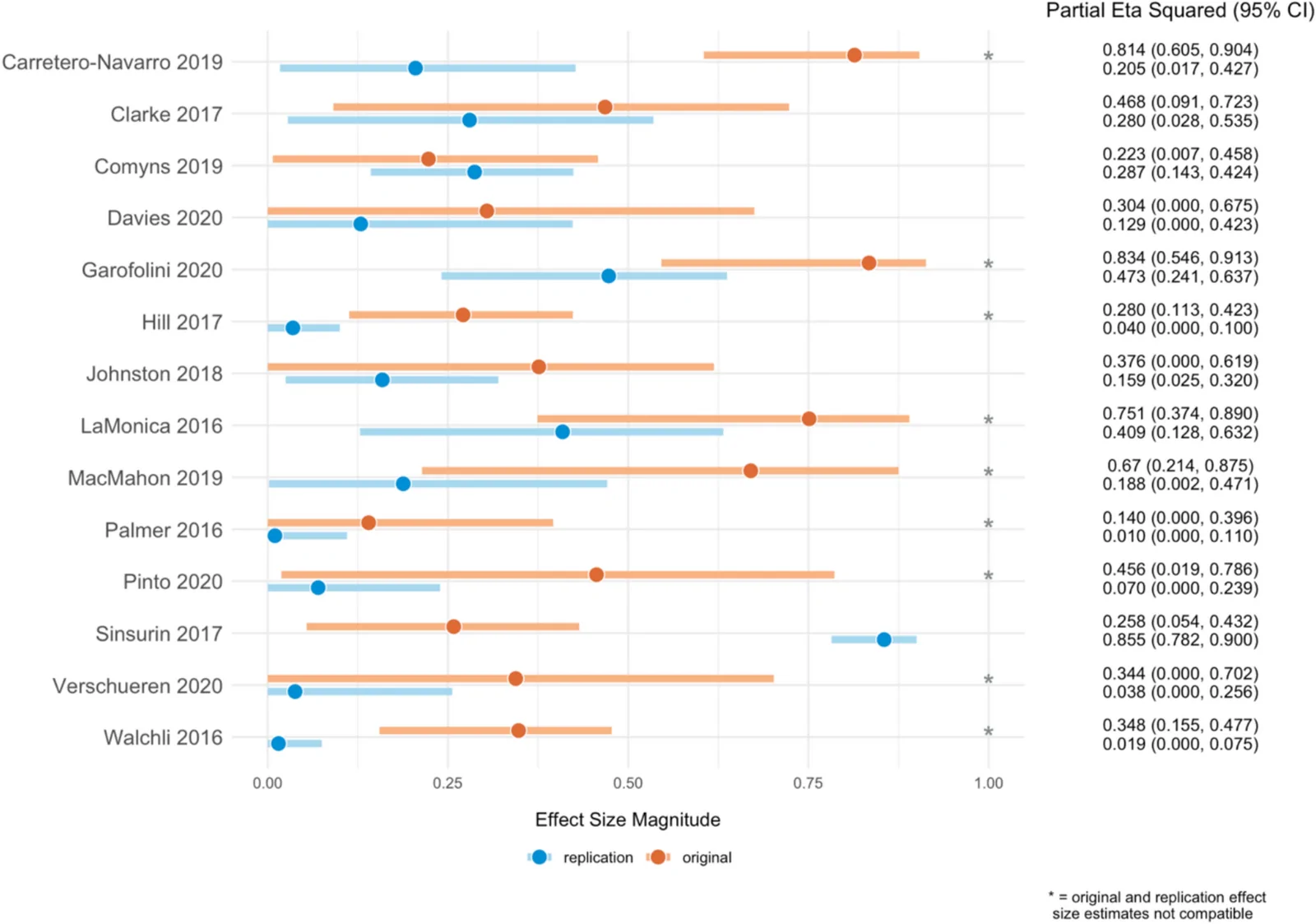
Plot showing original and replication partial eta squared magnitude and confidence intervals

For the 25 replication studies, 88% (*n* = 22) of the original effect sizes regressed to smaller values when replicated. For these replication studies (*n* = 25), the median percentage decrease in magnitude from the original to the replication effect size estimate was 75%. Our *Z*-test results showed that 64% (*n* = 16) of the replication effect size estimates were not statistically compatible with the original, and 36% (*n* = 9) were compatible (Figs. 1, 2).

Therefore, as per the classifications in Brandt et al. [35], 28% (*n* = 7) of the replications were successful, 36% (*n* = 9) were informative failures to replicate, 28% (*n* = 7) were practical failures to replicate and 8% (*n* = 2) were inconclusive (Tables 5, 6).

**Table 5:**
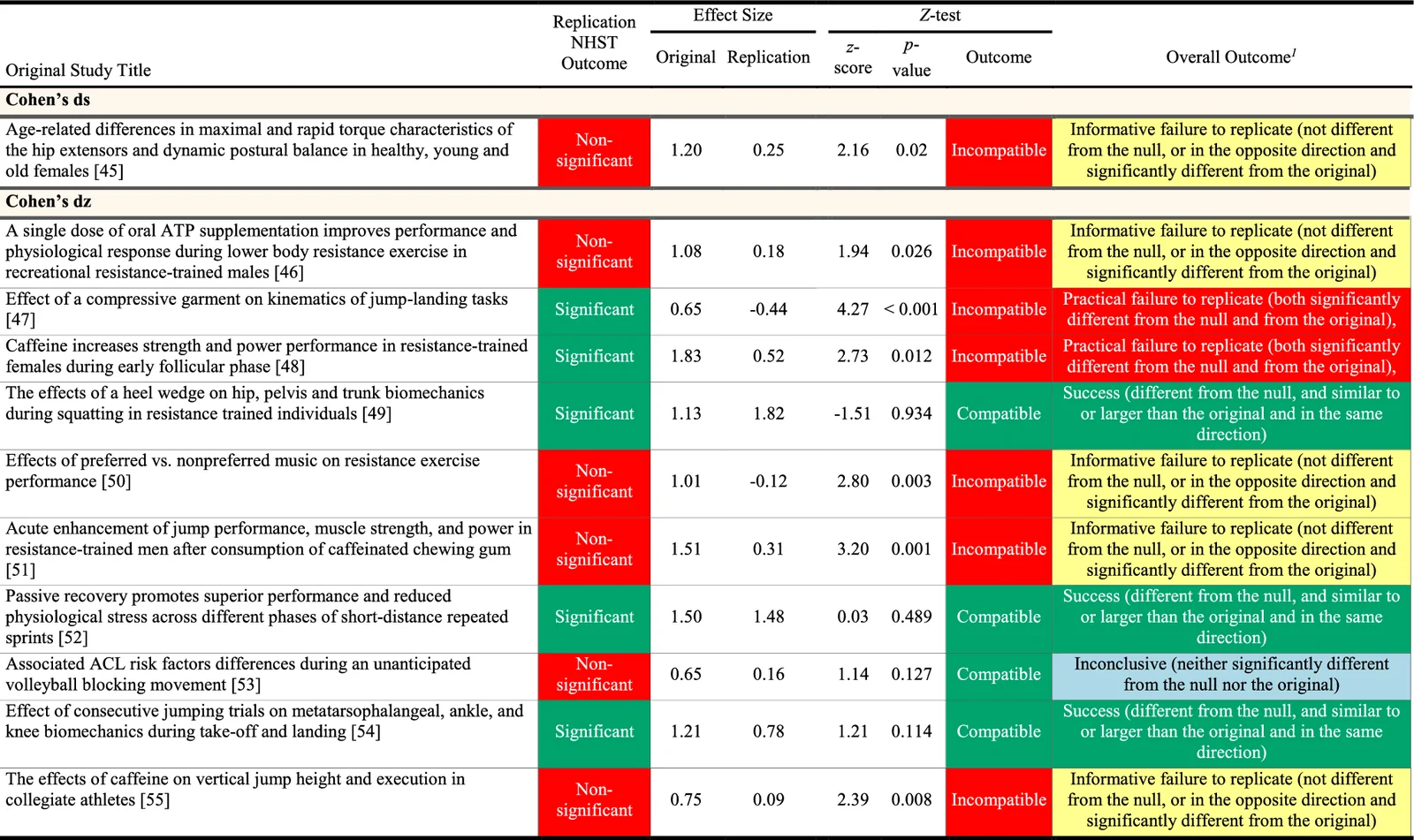
Overall replication outcomes and interpretations (Cohen’s *d*)

*ACL* anterior cruciate ligament, *ATP* adenosine triphosphate

^a^Brandt et al. [35]

**Table 6:**
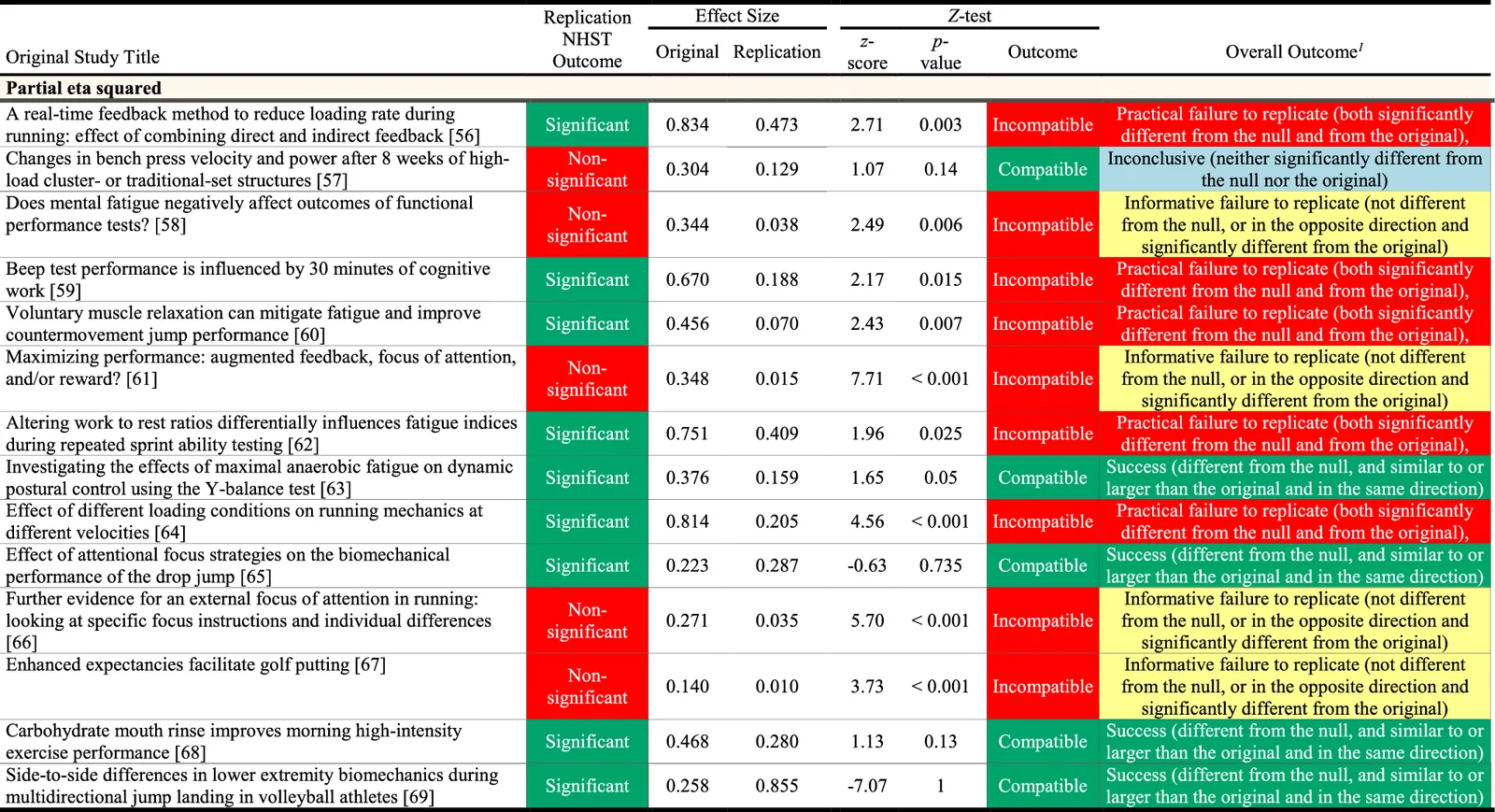
Overall replication outcomes and interpretations (partial eta squared)

^a^Brandt et al. [35]

## Discussion

This study aimed to provide an initial estimate of the replicability of sports and exercise science studies published between 2016 and 2021 in quartile 1 journals. We hypothesised that 61% of our studies could be expected to yield another significant effect given the previously published expected replication rate [22]. Considering our NHST-only results, 56% of the 25 analysed replication studies had a statistically significant *p*-value similar to the original studies. Yet, the effect size comparison via the *Z*-tests provides more context; only 36% of replication and original effect size estimates were compatible. When considering overall outcomes where they had both a significant NHST outcome and compatible effect sizes [35], 28% of replications were successful.

This pioneering project in sports and exercise science was the first large replication project in the field and the first to publish a transparent, randomised protocol for minimising bias in the selection of studies to replicate. The empirical data support subjective concerns about replication, reproducibility and transparency in sports and exercise science, as 78% of surveyed researchers believe there is a replication and reproducibility crisis in our field [28]. It also supports previous research implying we should have a healthy scepticism of our published literature, as it potentially includes a substantial number of false-positive findings [20]. Therefore, we echo calls for an immediate need to increase research transparency in our field [23, 71]. Many of the current research practices in sports and exercise science potentially contributed to the low replication rate e.g., publishing an excess of significant findings [20, 22, 25], using small sample sizes [21, 43, 72], poor reporting practices [22] and stating vague hypotheses [20], which will be discussed. Although issues with the current research practices have been identified already, replication has historically been undervalued in our field, and replication attempts are rare [20]. Perhaps the empirical data and low replication rate here will finally be the catalyst for change that is sorely needed in sports and exercise science.

The decrease in the magnitude of our replicated effect sizes from the originally reported effect sizes is a major concern. Replication effect sizes are expected to regress to their true values, as many original effect sizes tend to be inflated due to small sample sizes and publication bias [43]; these sample sizes are more likely to be affected by variation in the sampled data and other moderators (noise) even when designing replication studies close to the original [73]. We found that 88% of our replication effect sizes decreased in magnitude compared with the original effect sizes, with the median percentage decrease in magnitude from the original to the replication effect size estimate equal to 75% for all of the replication effect sizes (median of 77% for Cohen’s *d*_z_, 79% for Cohen’s *d*_s_ and 65% for partial eta squared). We expected some effect size regression, as the Reproducibility Project: Psychology reported the magnitude of the replication effect size estimates was approximately half that of the original effect size estimates [7]. However, the reduction in the magnitude of effect sizes was much larger in our study. The reported use of small sample sizes and the estimated low statistical power in sports and exercise science indicated that many effects might be much smaller in magnitude than originally published [21, 74]. The mean sample size was 17 participants across the original studies selected for replication, and most of the original effect sizes were considered large as per typical effect size threshold guidelines (all of the partial eta squared values were ≥ 0.14, and 73% of Cohen’s *d* values were > 0.8). Therefore, given the magnitude of the original effect size estimates and the original sample sizes, it is unsurprising that 64% of replication effect sizes were smaller than the original and were statistically incompatible. However, the magnitude of the difference in the effect size estimates was substantial, and this should be considered when using a published effect size in a power analysis for a subsequent study. Otherwise, statistical power based on this point estimate will be much lower than intended [36]. This regression towards smaller values also affects meta-analyses, as inflated effect sizes will impact the data presented. Sports and exercise scientists should therefore assume a large degree of uncertainty in published effect size estimates rather than assuming they are fixed or certain since most replication studies indicated substantial reductions in the effect size compared with the original.

Assessing the variability and uncertainty of published effect size estimates would be easier when the effect size and their confidence intervals are fully reported. Unfortunately, we had to compute the appropriate standardised effect size for the study design for 16 original studies, and 5 of these were conservatively estimated using the reported statistical information. Overall, 68% of the original studies reported some type of effect size, and this is a slightly lower reporting rate than the 79% reported by Twomey et al. [20], although they had a much larger sample size of 300 articles. Additionally, only 16% of the original studies here reported confidence intervals for either the standardised or unstandardised effect. All sports and exercise science researchers should fully report effect sizes and their confidence intervals (or, at minimum, the standard errors of the stated effect size) [75, 76]. This provides crucial information about the magnitude and uncertainty of observed effects, enabling readers to fully evaluate the data [17].

Issues with the reporting of effect size estimates also extended to reporting issues with the NHST framework itself. *F*-tests and *t*-tests are common statistical tests in sports and exercise science, but these tests are littered with errors in the literature [27]. For example, in one of the original studies herein, the analysis was reported as a two-way repeated measures ANOVA, but it was clear from the experimental design that the authors had conducted a mixed ANOVA. In terms of reporting quality, we found it to be poor, with only 48% reporting the test statistic and 36% reporting associated degrees of freedom. The lack of reporting of this information directly impacted the ability to evaluate methodological quality effectively [16], and this is a wider issue in sports and exercise science [77]. The lack of reporting of this critical information is worrisome considering the frequent use of frequentist statistical tests and the importance of such statistical tests to the study’s interpretation. Human error is also a factor, as many researchers, including those in the sports and exercise science field, undertake data analysis themselves without consulting other authors or statisticians [26]. The issues related to replication could be mitigated if researchers made their data, code and materials publicly available for other researchers to evaluate, which would assist with error correction in the long run. If data were made openly available, authors would be free to reduce the amount of cumbersome detail reported in the manuscript in favour of providing greater detail in online repositories of their data and analyses. Overall, the prevalence of errors and omitted information had a substantial impact on the ability to replicate studies within this project, and we encourage far better sharing of data and analyses in the future.

Our focus on replicating statistically significant findings for this project [34] was partly due to the high publication rate of significant findings in our field; Büttner et al. [24] reported an 82% positive result rate for sports medicine and physiotherapy, and Twomey et al. [20] reported an 81% for sports and exercise science. These rates could hypothetically be true if studies were appropriately planned to thoroughly inform hypotheses, the proportion of true hypotheses amongst all tested hypotheses was high, and the statistical power for each study was high. Yet, the studies would need more than 90% statistical power if all hypotheses tested were true to make this rate plausible [78]. Although many other questionable research practices and statistical errors can inflate the percentage of statistically significant effects, the high positive results rate is mostly likely facilitated by selectively reporting or “cherry-picking” desirable results, typically those results where* p* < 0.05, for maximum impact and publication potential i.e., publication bias. Publication bias is an observed phenomenon in sports and exercise science [22, 25] and is perpetuated by both researchers and journal publishers [28]. Consequently, publication bias could partially explain the low replication rate in this project. The replication of a study is difficult in cases of selective reporting or publication bias, as the original *p*-value might have passed the significance threshold at the upper tail of the* p*-value distribution [79]. Thus, a replication study with higher statistical power results in a non-significant *p*-value, thereby, non-replication. Publication bias in our field has possibly resulted in a published literature body of overinflated effect size estimates which regress by a median of 75% when replicated. However, replication is not normally attempted in sports and exercise science, and published claims are then “canonized” [80], leading to an accumulation of false claims in the literature that are considered irrefutable facts. When we combine this phenomenon with the inability to self-correct the literature because of poor data sharing, we likely create a knowledge base that fails to progress meaningfully.

This replication project sought to assess the validity of published findings in sports and exercise science, recognising that replication can either corroborate robust results or highlight those in need of re-evaluation. However, a study’s validity is often jeopardised before statistical analysis even begins due to flawed study design and the absence of a well-formulated hypothesis [27]. A key principle of NHST is the statements of the null hypothesis and alternative hypothesis, where the alternative hypothesis is sufficiently detailed to make it statistically falsifiable rather than just a complement of the null hypothesis [81]. Yet, falsifiability is rarely considered in hypothesis testing within sports and exercise science, as a substantial proportion of studies fail to formulate testable hypotheses and proceed to use NHST anyway [20]. In fact, hypotheses are often so vaguely stated in our field that any result could be spun to support the hypothesis due to researcher flexibility [20, 24, 82]. While any dataset can yield a significant finding, it may be a false positive, difficult to replicate and not representative of a true phenomenon [83]. Unfalsifiable original hypotheses may contribute to the low replication rate observed in this project. Additionally, such hypotheses make non-replications difficult to interpret, hindering knowledge advancement and theory development. The misinterpretation of the logic of hypothesis tests, their assumptions and the explicit control of type 1 and type 2 errors highlights a lack of understanding of experimental design and statistical practices amongst sports and exercise science researchers. Despite the field-wide acknowledgement of this [28], we rarely seek the help of statisticians [17] and continue to undertake flawed inferences, resulting in subsequently flawed published claims [84].

The universal norms of science include openness and rigour; therefore, sharing analytical materials (i.e., open data and code) is an essential step of an open and transparent scientific process [85], which we have already mentioned. Again, we want to emphasise that this practice allows researchers or reviewers to “trust but verify” [86], identify and correct errors, and facilitate secondary data analysis to update statistical models or extend knowledge of the topic under investigation [87]. This also helps facilitate meta-analysis and other evidence-synthesis endeavours [88]. However, most sports and exercise science manuscripts do not include open data and code. Borg et al. [23] observed that only 4% of 299 studies shared data, and no study shared any code or syntax related to the statistical analysis. Another team reported less than 1% of studies shared data [20]. The data sharing rate for this study was slightly higher albeit with a much smaller sample; we contacted 156 original authors for raw data and 14% shared original data, while 24% of the 29 completed replications shared data. When the original authors did not respond to requests for deidentified raw data, we asked them for complete statistical test details i.e., *t*-values or *F*-values, degrees of freedom, mean differences, etc., so that we could more accurately estimate effect sizes. None of the original authors provided this information. The level of data sharing in sports and exercise science appears to be considerably lower than in other fields [89, 90]. Data concealment can facilitate poor statistical analyses, manipulation, or selective reporting of results and a higher rate of statistical errors [89]. Although there can be concerns about data sharing, there are solutions to this [23]. There is also a need for a culture change to normalise that errors are part of the scientific process and should be communicated to original authors respectfully [91], but this can be delicate.

Lastly, the field of sport and exercise science faces unique challenges in advancing scientific knowledge due to its comparatively limited funding relative to other biomedical disciplines. This resource constraint magnifies the importance of research quality and methodological rigour. Enhancing transparency in research practices and eliminating questionable methodologies are not merely beneficial but essential for maximising the impact of available funding. By implementing more robust research practices, the field can ensure that limited resources are optimally utilised to generate reliable, replicable findings that meaningfully advance our understanding of human performance.

## Limitations

Our methodology stated that we aimed for 95% power, but it is far from realistic to assume we achieved this. In many of the replication studies we doubled the original sample size, and this undoubtedly led to underpowered replication studies; e.g., if an original study had a *p*-value of 0.03, the replication power is estimated to be only 50% when using this method [92]. In addition to the statistical power being less than intended in the replication studies, there are other limitations in this project. More than half of the studies in this pool did not replicate, and although we aimed to provide an initial estimate of the replicability of sports and exercise science research, this is certainly not a representative estimate. Firstly, 25 studies from the thousands of published sports and exercise science research could never possibly result in accurate estimates of replicability for the field. Secondly, much information had to be conservatively estimated e.g., original effect size estimates or test statistics (*t*-values or *F*-values) for analyses. This is not necessarily a limitation of the replication project itself—if we only selected original studies with all available information, this might have biased us towards higher-quality studies with better reporting—yet, it does affect the overall outcomes. When we had to conservatively estimate effect sizes where they were not reported in the original study, we calculated the smallest plausible effect size on the basis of the reported relative *p*-value, meaning that the original effect may have been larger than we estimated. Additionally, the comparison between ANOVA effect sizes i.e. partial eta-squared, is only an approximation based on Fisher’s *z* transformation, which simulations indicated preserved type 1 error well. Therefore, the outcome of the *Z*-tests here may be more conservative than the true original values.

In our selection protocol, we stated that we would not try to improve any methods so that we could attempt to replicate the same theoretical dimensions of the original study. This potentially means we could have taken poorly designed original studies and created poorly designed replication studies (Table 7). In some cases, a lack of methodological details in the original studies also made some aspects of the replication questionable. Critics will view this as a waste of resources, but the intention of our project was not to update knowledge on particular theories but to “simply” replicate what was published, in a way that minimised bias and maximised representativeness of applied sports and exercise science research.

**Table 7:**
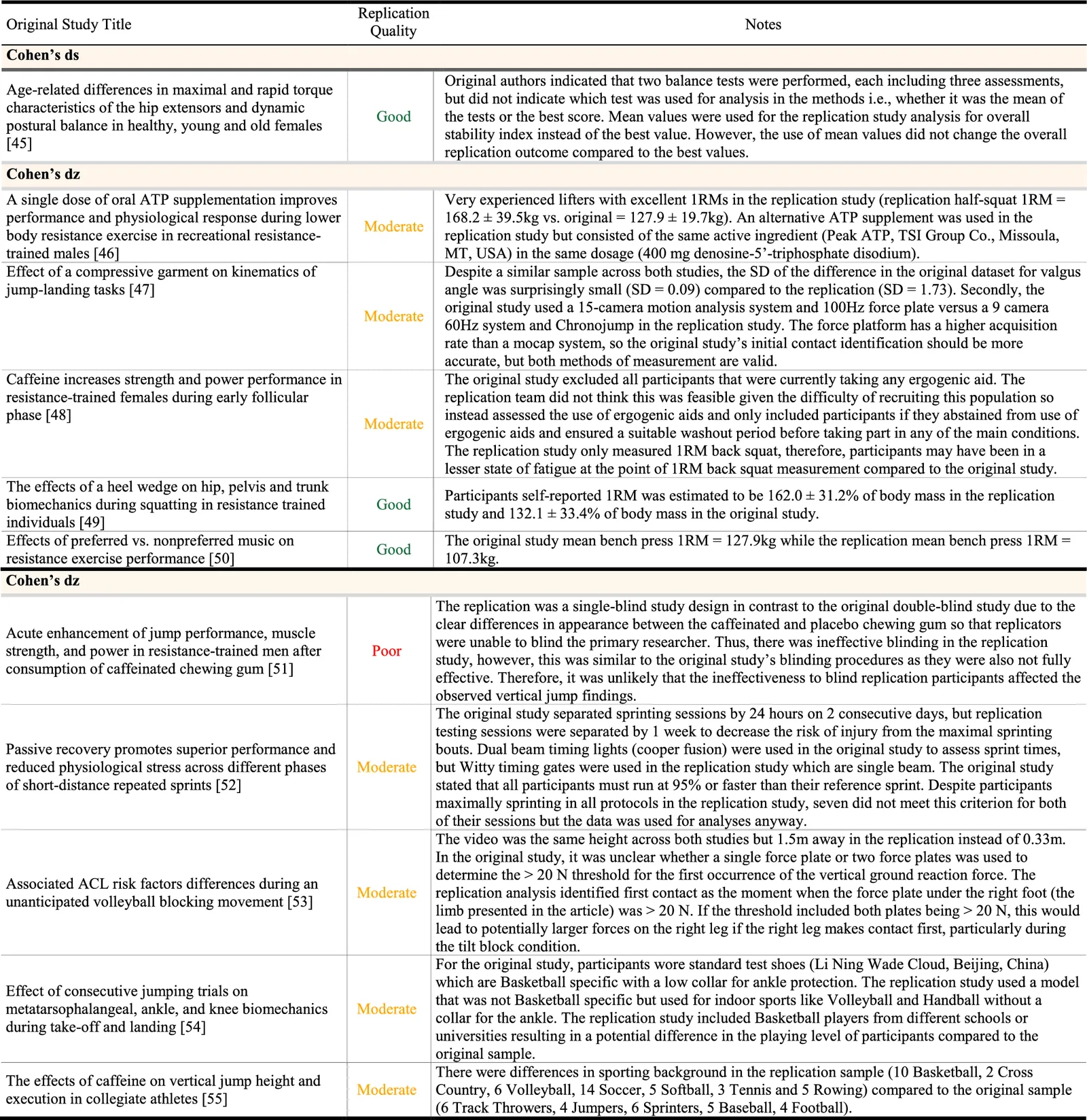

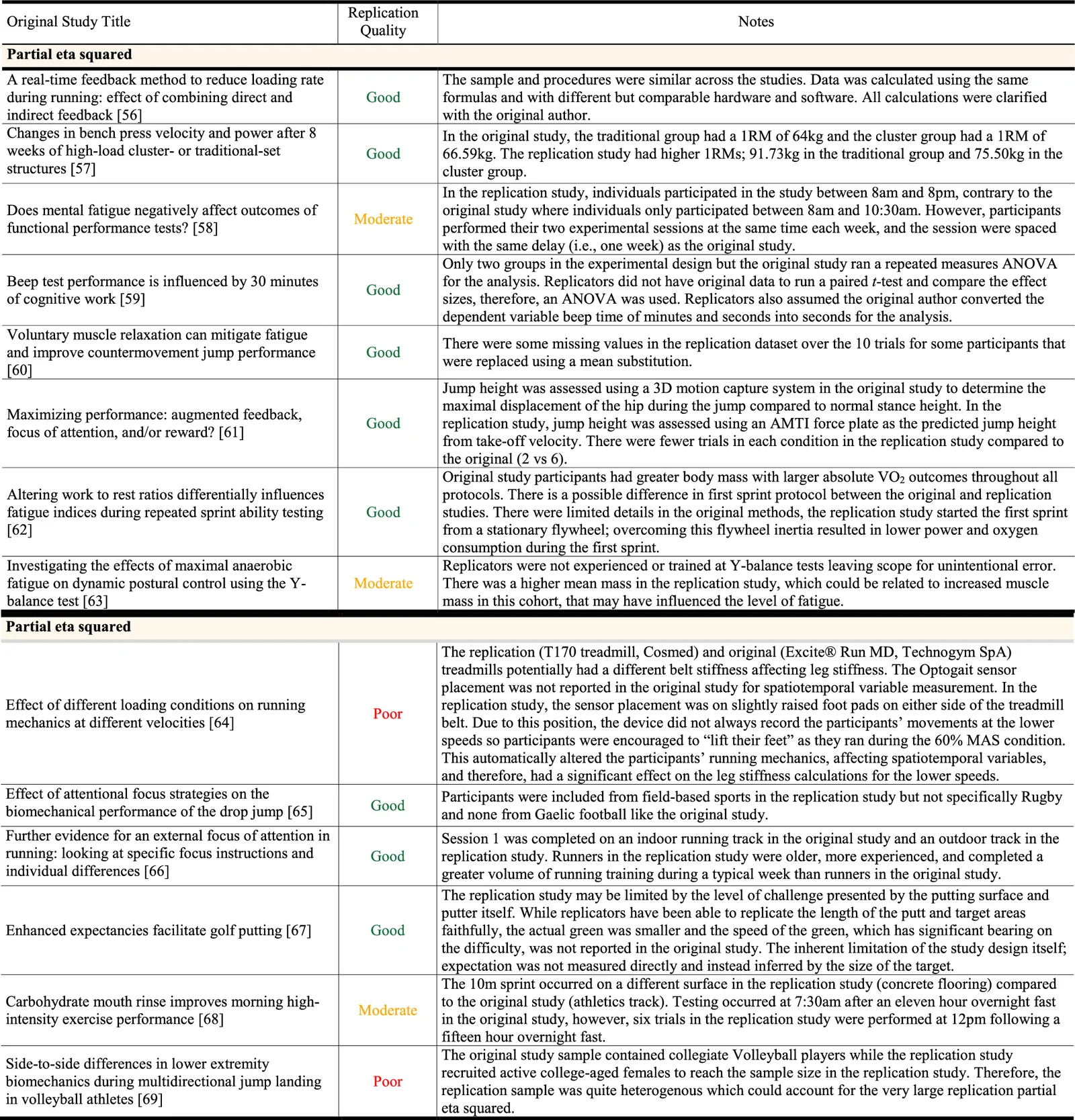
Subjective assessment of replication quality

*3D* three-dimensional, *MAS* maximal aerobic speed, *RM* repetition maximums, *SD* standard deviation, *VO*_*2*_ oxygen consumption

## Future Recommendations

In summary of the current state of published sports and exercise science research, we know that statistical and methodological errors and lack of reporting transparency are prevalent in sports and exercise science [27, 74, 93]. Over three-quarters (78%) of surveyed sports and exercise science researchers already believe there is a replication and reproducibility crisis in sports and exercise science and that poor experimental design, insufficient mentoring, publishing pressure and selective reporting are the factors contributing most towards failed replications [28]. In addition, we know that we overestimate our level of statistical expertise and that a better understanding of study design, and the use of different statistical techniques to analyse data, would improve reproducibility and replicability [16, 28]. Therefore, there are many avenues that we can improve in the future.

As highlighted previously, discerning the “truth” between an original and replication study solely on the basis of their results is inherently fraught. This limitation is not specific to our project but rather a fundamental reality of research. It remains exceedingly difficult to definitively disprove a theory or claim solely on the basis of a single, divergent replication [94]. Therefore, we must evaluate the quality and rigour of our published claims on the basis of the study characteristics rather than placing irrational privilege in the chronological order of studies [95]. Original studies should not be prioritised over replication studies, and a replication study cannot overturn the original study results; rather, the focus should be on the accumulation of evidence rather than each study in a standalone manner. If a replication and original study differ, an auxiliary hypothesis can be formulated to expand a theory: i.e., “sophisticated falsification” [96]. If multiple replications are undertaken and more “falsification” arises (i.e., diagnostic evidence that does not support the claim), a “strategic retreat” of the original claim is warranted [94]. Therefore, non-replications can be informative by identifying boundary conditions for a claim and leading to the generation of new or reformulated hypotheses [97]. Furthermore, sports and exercise science researchers must embrace intellectual humility and acknowledge the inherent uncertainty in their work [98]. Transparency about flaws and limitations fosters humility, while concealing them breeds overconfidence, arrogance and intellectual fragility [98]. Achieving the ideals of quality and rigour over mere outcomes requires time and patience, and while crucial, replicability is but one facet of high-quality science. To ensure the long-term health of our field, we can take immediate action. Table 8 provides a roadmap, offering recommendations and resources to guide the implementation of these practices into our research endeavours.

**Table 8:** A list of recommendations and resources for improving research practices in sports and exercise science

Action	Why?	How?
Justify the sample size	Power, precision and sample size estimation in sport and exercise science research [21]: https://doi.org/10.1080/02640414.2020.1776002	Sample size justification [99]: https://doi.org/10.1525/collabra.33267
State a clear hypothesis	Degrees of freedom in planning, running, analysing and reporting psychological studies: a checklist to avoid *p*-hacking [82]: https://doi.org/10.3389/fpsyg.2016.01832	Why hypothesis testers should spend less time testing hypotheses [81]: https://doi.org/10.1177/1745691620966795
Pre-register the hypothesis and analyses	Preregistration is hard and worthwhile [100]: https://doi.org/10.1016/j.tics.2019.07.009	https://osf.io/registries https://sportrxiv.org/index.php/server
Consider a registered report	Moving sport and exercise science forward: a call for the adoption of more transparent research practices [71]: https://doi.org/10.1007/s40279-019-01227-1	https://www.cos.io/initiatives/registered-reports Sports and exercise science example: https://doi.org/10.51224/cik.v1i3.43
Conduct appropriate statistical analysis	Improving your statistical inferences [101]: https://lakens.github.io/statistical_inferences/	PsyteachR open source textbooks: https://psyteachr.github.io
Fully report all statistical results	Replication concerns in sports and exercise science: a narrative review of selected methodological issues in the field [16]: https://doi.org/10.1098/rsos.220946	Publication bias, statistical power and reporting practices in the *Journal of Sports Sciences*: potential barriers to replicability [22]: https://doi.org/10.1080/02640414.2023.2269357
Report effect sizes and their magnitudes	Calculating and reporting effect sizes to facilitate cumulative science: a practical primer for *t* tests and ANOVAs [70]: https://doi.org/10.3389/fpsyg.2013.00863	Guide to effect sizes and confidence intervals [102]: https://doi.org/10.17605/OSF.IO/D8C4G TOSTER package: https://aaroncaldwell.us/TOSTERpkg/articles/SMD_calcs.html
Minimise statistical errors	Wish list for improving the quality of statistics in sports science [17]: https://doi.org/10.1123/ijspp.2022-0023 Call to increase statistical collaboration in sports science, sport and exercise medicine and sports physiotherapy [26]: https://doi.org/10.1136/bjsports-2020-102607	Ten common statistical errors from all phases of research, and their fixes [27]: https://doi.org/10.1002/pmrj.12395 *p*-checker: https://shinyapps.org/apps/p-checker/
Make your code and data open	Comment on: ‘Moving sport and exercise science forward: A call for the adoption of more transparent research practices’ [23]: https://doi.org/10.1007/s40279-020-01298-5	OSF: https://osf.io GitHub: https://github.com

*OSF* Open Science Framework

## Conclusion

In the first collaborative sports and exercise science replication project, only 28% of studies were successfully replicated, and there was a substantial regression of the reported effect size estimates. The low replication rate is potentially caused by poor research practices in our field e.g., publication bias towards significant findings [20, 22, 25], the use of small sample sizes [21, 43], poor reporting practices [22] and unfalsifiable, vague hypotheses [20]. Consequently, our current practices made it challenging to conduct this large replication project in sports and exercise science. The results of this project, in combination with previous research identifying issues in our field [16, 20, 22, 28], do not alleviate any concerns about the internal validity of sports and exercise science research and suggest a need to improve our research practices moving forward. To improve sports and exercise science research, we must make changes to our scientific process and culture, and we have recommended changes and provided a list of resources to assist with this. We hope that this preliminary outcome will excite a renewed vigour into conversations around research culture and current practices.

## Declarations

### Availability of data and material

Raw data, code and other supplementary materials are available at https://doi.org/10.17605/OSF.IO/SFBVA.
