# Correction: Estimating the Replicability of Sports and Exercise Science Research

**DOI:** 10.1007/s40279-025-02309-z

**Published:** 2025-09-11

**Authors:** Jennifer Murphy, Aaron R. Caldwell, Cristian Mesquida, Aera J. M. Ladell, Alberto Encarnación-Martínez, Alexandre Tual, Andrew Denys, Bailey Cameron, Bas Van Hooren, Ben Parr, Bianca DeLucia, Billy R. J. Mason, Brad Clark, Brendan Egan, Calum Brown, Carl Ade, Chiarella Sforza, Christopher B. Taber, Christopher Kirk, Christopher McCrum, Cian OKeeffe Tighe, Ciara Byrne, Claudia Brunetti, Cyril Forestier, Dan Martin, Danny Taylor, David Diggin, Dearbhla Gallagher, Deborah L. King, Elizabeth Rogers, Eric C. Bennett, Eric T. Lopatofsky, Gemma Dunn, Gérome C. Gauchard, Guillaume Mornieux, Ignacio Catalá-Vilaplana, Ines Caetan, Inmaculada Aparicio-Aparicio, Jack Barnes, Jake Blaisdell, James Steele, Jared R. Fletcher, Jasmin Hutchinson, Jason Au, Jason P. Oliemans, Javad Bakhshinejad, Joaquin Barrios, Jose Ignacio Priego Quesada, Joseph Rager, Julia B. Capone, Julie S. J. Walton, Kailey Stevens, Katie Heinrich, Kelly Wu, Kenneth Meijer, Laura Richards, Lauren Jutlah, Le Tong, Lee Bridgeman, Leo Banet, Leonard Mbiyu, Lucy Sefton, Margaux de Chanaleilles, Maria Charisi, Matthew Beerse, Matthew J. Major, Maya Caon, Mel Bargh, Michael Rowley, Miguel Vaca Moran, Nicholas Croker, Nicolas C. Hanen, Nicole Montague, Noel E. Brick, Oliver R. Runswick, Paul Willems, Pedro Pérez-Soriano, Rebecca Blake, Rebecca Jones, Rebecca Louise Quinn, Roberto Sanchis-Sanchis, Rodrigo Rabello, Roisin Bolger, Roy Shohat, Sadie Cotton, Samantha Chua, Samuel Norwood, Samuel Vimeau, Sandro Dias, Sissel Pedersen, Spencer S. Skaper, Taylor Coyle, Terun Desai, Thomas I. Gee, Tobias Edwards, Torsten Pohl, Vanessa Yingling, Vinicius Ribeiro, Youri Duchene, Zacharias Papadakis, Joe P. Warne

**Affiliations:** 1 School of Biological, Health and Sport Sciences, Technological University Dublin, Tallaght Campus, Blessington Rd, Dublin 9, Ireland; 2 Institute for Community Health Innovation, University of Arkansas for Medical Sciences Northwest, Springdale, AR 72762 USA; 3 Human-Technology Interaction Group, Eindhoven University of Technology, Eindhoven, The Netherlands; 4 Department of Health and Physical Education, Mount Royal University, Calgary, AB T3E 6K6 Canada; 5 Department of Physical Education and Sports, Universitat de València, València, Spain; 6 Laboratoire Motricité, Interactions, Performance, Le Mans Université, Le Mans, France; 7 Department of Kinesiology, California State University East Bay, Hayward, CA 94542 USA; 8 Department of Sport and Health, Solent University, Southampton, SO14 0YN UK; 9 Department of Nutrition and Movement Sciences, NUTRIM School for Nutrition and Translational Research in Metabolism, Maastricht University, 6211 LK Maastricht, The Netherlands; 10 School of Psychology, Ulster University, Coleraine, Co., Londonderry, BT52 1SA UK; 11 Department of Exercise Science and Athletic Training, Springfield College, Springfield, MA USA; 12 University of Canberra Research Institute for Sport and Exercise, University of Canberra, Bruce, ACT 2617 Australia; 13 School of Health and Human Performance, Dublin City University, Glasnevin, Dublin 9, Ireland; 14 School of Sport and Physical Activity, Sheffield Hallam University, Sheffield, UK; 15 Department of Kinesiology, Kansas State University, Manhattan, KS 66506 USA; 16 Department of Biomedical Sciences for Health, Università Degli Studi Di Milano, Via Mangiagalli, 31, 20133 Milan, Italy; 17 Department of Physical Therapy and Human Movement Science, Sacred Heart University, Fairfield, CT USA; 18 School of Sport and Exercise Science, University of Lincoln, Lincoln, LN6 7TS UK; 19 School of Health Sciences and Human Performance, Ithaca College, Ithaca, NY 14850 USA; 20 School of Human and Social Sciences, Buckinghamshire New University, High Wycombe, UK; 21 Development, Adaptation, Handicap-CARE Grand Est, Université de Lorraine, Nancy, France; 22 Department of Physical Therapy, The University of British Columbia, Vancouver, Canada; 23 Department of Kinesiology and Health Sciences, University of Waterloo, Waterloo, ON Canada; 24 Department of Physical Therapy, University of Dayton, Dayton, OH USA; 25 Institute of Psychiatry, Psychology and Neuroscience, Kings College London, London, UK; 26 Faculty of Kinesiology, University of Calgary, Calgary, AB T2N 1N4 Canada; 27 Department of Physical Medicine & Rehabilitation, Northwestern University, Chicago, IL USA; 28 Human Performance Laboratory and Motion Analysis Center, Department of Health Promotion and Clinical Practice, Barry University, Miami Shores, FL 33161 USA; 29 Specialist Facilities, Solent University, Southampton, SO14 0YN UK; 30 Institute of Sport, Exercise and Health, Division of Surgery and Interventional Science, University College London, London, UK; 31 Conservative and Rehabilitative Orthopaedics, Technical University of Munich, G80992 Munich, Germany

**Correction: Sports Medicine** 10.1007/s40279-025-02201-w

In this article, author names and affiliations were incorrectly written as Julia B. Capon, Gérome C. Gauchar, Margaret de Chanaleilles and Sheffield University (affiliation 14).

The correct author names and affiliations should have been: Julia B. Capone, Gérome C. Gauchard, Margaux de Chanaleilles, Sheffield Hallam University

The original article has been updated accordingly.

